# FDG-PET/CT imaging for tumor staging and definition of tumor volumes in radiation treatment planning in non-small cell lung cancer

**DOI:** 10.3892/ol.2014.1874

**Published:** 2014-02-12

**Authors:** YUANDA ZHENG, XIAOJIANG SUN, JIAN WANG, LINGNAN ZHANG, XIAOYUN DI, YAPING XU

**Affiliations:** 1 Department of Radiation Oncology, Zhejiang Cancer Hospital, Hangzhou, Zhejiang 310022, P.R. China; 2 Department of Radiology, Zhejiang Cancer Hospital, Hangzhou, Zhejiang 310022, P.R. China

**Keywords:** positron emission tomography/computed tomography, non-small cell lung cancer, radiotherapy, tumor volume

## Abstract

^18^F-fluorodeoxyglucose (FDG)-positron emission tomography (PET)/computed tomography (CT) has the potential to improve the staging and radiation treatment (RT) planning of various tumor sites. However, from a clinical standpoint, questions remain with regard to what extent PET/CT changes the target volume and whether PET/CT reduces interobserver variability in target volume delineation. The present study analyzed the use of FDG-PET/CT images for staging and evaluated the impact of FDG-PET/CT on the radiotherapy volume delineation compared with CT in patients with non-small cell lung cancer (NSCLC) who were candidates for radiotherapy. Intraobserver variation in delineating tumor volumes was also observed. In total, 23 patients with stage I-III NSCLC were enrolled and treated with fractionated RT-based therapy with or without chemotherapy. FDG-PET/CT scans were acquired within two weeks prior to RT. PET and CT data sets were sent to the treatment planning system, Pinnacle, through compact discs. The CT and PET images were subsequently fused by means of a dedicated RT planning system. Gross tumor volume (GTV) was contoured by four radiation oncologists on CT (GTV-CT) and PET/CT images (GTV-PET/CT). The resulting volumes were analyzed and compared. For the first phase, two radiation oncologists outlined the contours together, achieving a final consensus. Based on PET/CT, changes in tumor-node-metastasis categories occurred in 8/23 cases (35%). Radiation targeting with fused FDG-PET and CT images resulted in alterations in radiation therapy planning in 12/20 patients (60%) in comparison with CT targeting. The most prominent changes in GTV were observed in cases with atelectasis. For the second phase, the variation in delineating tumor volumes was assessed by four observers. The mean ratio of largest to smallest CT-based GTV was 2.31 (range, 1.01–5.96). The addition of the PET results reduced the mean ratio to 1.46 (range, 1.02–2.27). PET/CT fusion images may have a potential impact on tumor staging and treatment planning. Implementing matched PET/CT results reduced observer variation in delineating tumor volumes significantly with respect to CT only.

## Introduction

In locally advanced non-small cell lung cancer (NSCLC), definitive radiation treatment (RT) provides improved disease control and survival rates through high-dose radiation and concurrent administration of systemic drugs. The recent introduction of sophisticated technology, such as three-dimensional conformal RT (3DCRT), promises to improve the cost/benefit ratio of therapy further.

Currently, the issue of precisely identifying the tumor volume remains open; tumor dose escalation and sparing of normal tissue requires the extent of the disease to be precisely identified in each patient. As a result, there is considerable interest in investigating functional imaging, including positron emission tomography (PET) particularly with ^18^F-fluorodeoxyglucose (FDG), that provides improved tumor staging, delineation of the target volume, identification of the treatment response and detection of recurrence for a wide range of solid cancer types (1–3). However, due to a lack of anatomical information, a careful correlation must be made between FDG-PET and structural images in order to precisely localize the tumor. A range of image registration strategies allows FDG-PET images to be directly incorporated into computed tomography (CT) images. This allows the complementary strengths of functional (PET) and structural (CT) imaging to be co-registered in a single image set.

A review of the use of PET/CT to define the tumor volume in the RT planning of NSCLC identified 15 previously published studies (4). Only 7 of those included >21 patients and only limited studies compared intraobserver variation. The current study is an important contribution that brings under discussion two critical issues with respect to the effect of fused PET/CT on the delineation of gross tumor volume (GTV) in the RT planning of NSCLC patients. These issues are with regard to what extent PET/CT changes the target volume and whether PET/CT reduces interobserver variability in target volume delineation

## Materials and methods

### Patient characteristics

Between January 2006 and December 2007, 23 consecutive patients with NSCLC were referred for CRT, planned using a fully integrated PET/CT device. All patients were selected for RT, and informed written consent was obtained following the rules of the Zhejiang Cancer Hospital (Hangzhou, China). The clinical stage of the disease varied between I and IIIB, and the median age was 63 years (range, 43–76 years). Patients with a Karnofsky performance status of <80, evidence of distant metastases at initial staging or a requirement for surgical procedures were excluded. The clinical characteristics of the included patients are summarized in Table I. All patients underwent a routine workup*,* including a clinical examination, fiber endoscopy, contrast-enhanced CT of the chest, liver ultrasound and brain magnetic resonance imaging. The clinical stage was retrospectively defined according to the 2009 Union for International Cancer Control classification (5). No patients were candidates for curative surgery. However, 15 patients were candidates for combined RT and platinum-based chemotherapy and five patients for RT alone. In addition, three patients were diagnosed with metastatic disease based on FDG-PET/CT and received palliative chemotherapy. This study was approved by the Ethical Review Committee, Zhejiang Cancer Hospital (Hangzhou, China). Recommendations from the Helsinki Declaration for biomedical research involving human subjects were followed.

### Image acquisition and fusion

All patients underwent PET/CT simulation in the supine position, while immobilized with a customized thermoplastic mask and using the Biograph 16 HI-REZ PET/CT scanner (Siemens Healthcare, Hoffman Estates, IL, USA). The PET component was a high-resolution scanner with a spatial resolution of 4.7 mm and no septa, thus allowing 3D-only acquisitions. The CT component used was the Somatom Sensation 16-slice CT (Siemens Healthcare). The CT scanner was used for attenuation correction of the PET results and for localization of FDG uptake in the PET images. All patients were advised to fast for ≥6 h prior to PET/CT examination. Following injection of ~5 MBq FDG per kg of body weight, the patients were rested for a period of ~60 min in a comfortable chair. Emission images ranging from the proximal femur to the base of the skull were acquired for 2–3 min per bed position. The field of view was 50 cm, with a matrix of 512×512 pixels for CT and 128×128 pixels for PET. The processed images were exhibited in coronal, transverse and sagittal planes. Following image acquisition, PET and CT data sets were sent to the treatment planning system, Pinnacle system (Philips Medical Systems, Milpitas, CA, USA), through compact discs. The CT and PET images were subsequently fused by means of a dedicated RT planning system image fusion tool based on a mutual information algorithm.

### Tumor staging and target volume delineation

The clinical staging was analyzed by comparing the PET/CT with the CT observations alone. For clinical purposes, the PET image was always considered as additional information to the CT image for tumor staging or target contouring for treatment planning.

The target volumes were outlined by two radiation oncologists with specific experience in lung cancer management according to the guidelines to contour the GTV of the Radiation Therapy Oncology Group 0515 (6). The oncologists were not blinded to each other and worked together to outline the contours, achieving a final consensus. The GTV-CT was defined per CT result as only the gross tumor and any lymph nodes with a cross-sectional diameter of ≥1 cm. Lung window settings were used to contour the primary lesion, and soft tissue windows were used for contouring the lymph nodes. GTV-PET/CT was then defined using fully fused PET/CT imaging as the PET-visualized enhancement of the gross tumor and any lymph node with an average standard uptake value (SUV) of ≥2.5 (regardless of any deficiency in adequate nodal size criteria for malignancy as visualized by CT images alone) or any lymph nodes with a cross-sectional diameter of ≥1 cm on CT. The GTV-PET/CT included PET and CT information.

For the second phase, four independent observers were asked to contour and record the GTV-CT and GTV-PET/CT.

## Results

### Tumor staging

PET/CT imaging led to a change in the tumor-node-metastasis (TNM) stage of 8/23 cases (35%) compared with CT alone (Table II). T-stage changed in 4/23 cases (17%) and N-stage in 7/23 cases (30%).

### Target volumes

**Figure 1:**
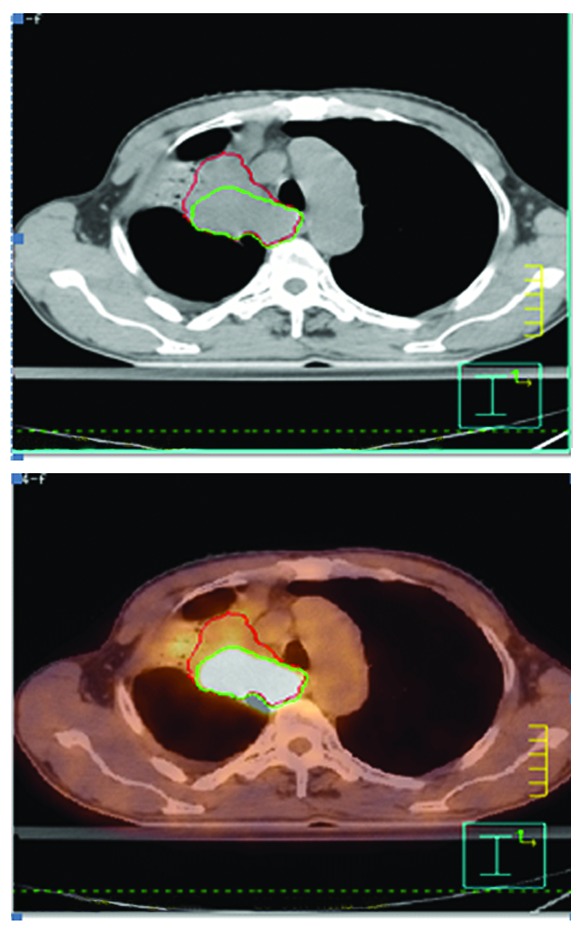
Alteration of gross tumor volume (GTV) by positron emission tomography/computed tomography (PET/CT) scan in one atelectasis patient. The red and green outlined areas indicate GTV based on the CT image and the PET/CT image, respectively.

**Figure 2:**
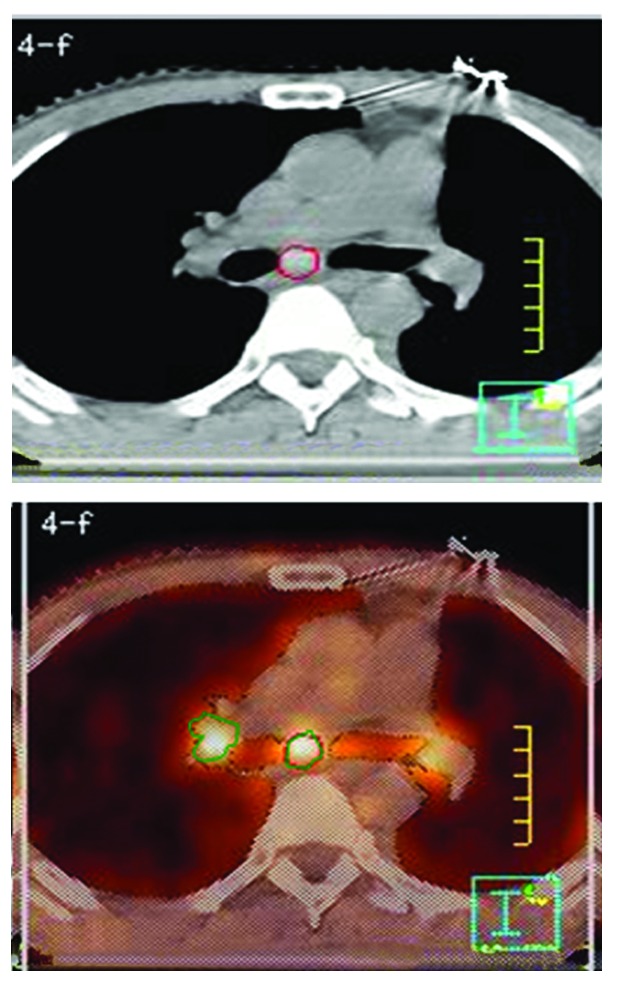
Alteration of gross tumor volume (GTV) by positron emission tomography/computed tomography (PET/CT) scan in one patient with unsuspected nodal disease. The red and green outlined areas indicate GTV based on the CT image and the PET/CT image, respectively.

Of the 20 patients who were not diagnosed with metastatic disease based on FDG-PET/CT planned with 3DCRT, PET/CT clearly altered the radiation therapy volume (>10%) in 12/20 patients (60%) in comparison with CT targeting, which were outlined together by two radiation oncologists. The analyzed volumes for all patients are reported in Table III. PET aided in the ability to distinguish tumors from atelectasis in all five patients with atelectasis. Atelectasis patients 3, 12, 19 and 20 were cases where the addition of FDG significantly reduced GTV. In atelectasis patient 16, the primary lesion was reduced, but positive mediastinal lymph nodes were detected by PET/CT. Overall, no change in GTV was identified. Unsuspected nodal disease was detected by PET/CT in all 10 patients (two patients with atelectasis of the lung). Patient 10 exhibited a separate tumor focus detected within the same lobe of the lung, and GTV was also increased. The alteration of GTV by PET/CT scan is demonstrated in Fig. 1 in one atelectasis patient and in Fig. 2 in one unsuspected nodal disease patient.

### Magnitude of interobserver variability

For the second phase, the intraobserver variation in delineating tumor volumes was assessed by four observers. The concordance in GTV of the four observers was increased by the use of PET/CT. The mean ratio of largest to smallest CT-based GTV was 2.31 (range, 1.01–5.96). The addition of the PET results reduced the mean ratio to 1.46 (range, 1.02–2.27). The comparison of GTV-CT and GTV-PET/CT for each patient and observer is reported in Table IV.

**Figure 3:**
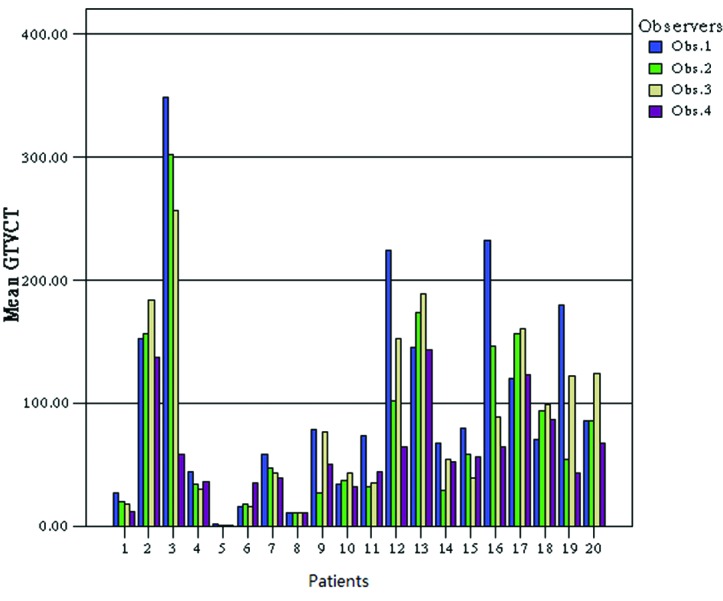
Comparison of GTV according to CT (GTV-CT) delineated by four observers. GTV, gross tumor volume; CT, computed tomography.

**Figure 4:**
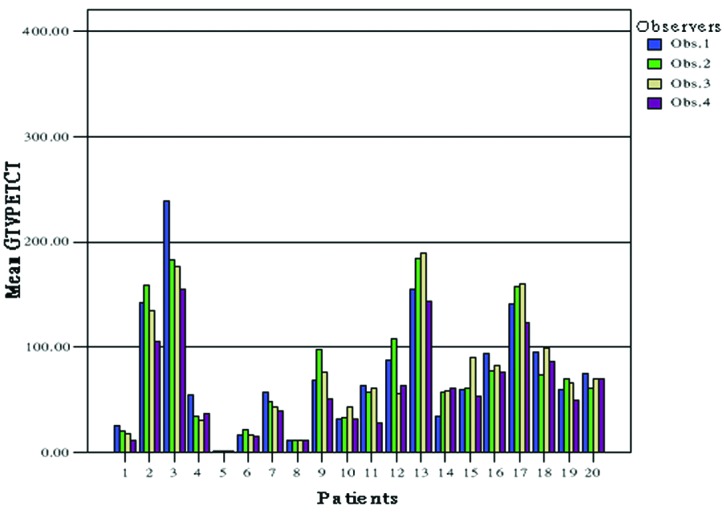
Comparison of GTV according to PET/CT (GTV-PET/CT) delineated by four observers. GTV, gross tumor volume; PET/CT, positron emission tomography/computed tomography.

The variation in GTV between observers using CT alone is illustrated in Fig. 3, whereas the concordance in delineating GTV and PTV by PET/CT is demonstrated in Fig. 4.

## Discussion

The use of FDG-PET/CT prior to treatment has gained interest in the radiation oncology community in association with a potential improvement in tumor staging, the optimization of treatment strategy and an improved delineation of target volume (7). Preliminary results for numerous tumor locations, including the head and neck, lung, esophagus, rectum, anal canal and pancreas, have been reported in a number of previous literature studies (8–12). Certain studies concerning the advantage of PET/CT fusion have been previously reported for radiation planning of patients with NSCLC (13–15).

In the current study, based on PET/CT, changes in TNM stage occurred in 8/23 cases (35%). Similar results were reported by Bradley *et al* (16), where 26 patients were enrolled and PET/CT fusion was found to alter the TNM categories in 31% of patients (8/26). In the present study, three patients were diagnosed with metastatic disease based on FDG-PET and received palliative radiation therapy. To date, it appears that the best benefit from FDG-PET in NSCLC is the evaluation of the cancer extent. FDG-PET/CT identifies patients with tumor spread not detected by standard methods in ~20% of the cases (17).

In the current study, the effect of PET/CT on the delineation of GTV was evaluated considering the PET information in addition to that of CT. PET aided the ability to distinguish tumors from atelectasis in all five patients with atelectasis. Unsuspected nodal disease was detected by PET in 10 patients, and one patient exhibited a separate tumor focus detected within the same lobe of the lung. Previous studies have shown that fused PET and CT alters ~50% of the GTV delineation compared with CT targeting alone, as observed by visual evaluation or using specific mathematical algorithms, such as a fixed SUV or threshold (18,19). The presence of atelectasis, which appears easier to differentiate from tumors with the use of combined PET/CT information, often leads to a significant decrease in GTV (20–24). Since routine elective nodal irradiation is often omitted in NSCLC, accurate identification of the involved nodal areas is pivotal in modern treatment planning with curative intent. A number of previous studies have shown significant changes in GTV volume due to the inclusion or exclusion of nodal areas compared with CT-alone GTV (25–27). In a prospective clinical study, De Ruysscher *et al* (28) evaluated the patterns of recurrence following FDG-PET-based selective mediastinal node irradiation in 44 patients with NSCLC. The rationale of the study was the higher diagnostic accuracy of FDG-PET on mediastinal lymph node areas. Only one case of nodal recurrence was reported from the 44 patients selectively irradiated on the FDG-avid areas of the mediastinum.

In the second part of the present study, the results showed that FDG-PET reduced the ratio of largest to smallest GTV in the majority of cases (14/20). The concordance in treatment planning of the four observers was increased by the use of PET/CT. The mean ratio of largest to smallest CT-based GTV was 2.31 (range, 1.01–5.96). The addition of the PET results reduced the mean ratio to 1.46 (range, 1.12–2.27). Atelectasis patients 3, 12, 16, 19 and 20 were cases where the addition of FDG evidently reduced interobserver variability. For patient 19 (Fig. 1), it was difficult to distinguish atelectasis of the lung from the tumor using CT alone; FDG clarified the location of the tumor and aided the determination of a consistent boundary. For patient 15, FDG aided the ability of the four observers to achieve a more consistent conclusion concerning the presence of nodal disease. In the majority of cases where the addition of FDG appeared to increase interobserver variability, the increase was minor. However, in patient 11, the addition of FDG did not significantly reduce variability. The SUV of FDG uptake was <2.5 in the lymph nodes with a cross-sectional diameter of 1 cm on CT, which three of the four observers interpreted as being positive lymph nodes. However, the fourth observer interpreted the FDG uptake in the lymph nodes as not indicating tumor metastasis.

The majority of previous studies, as well as the present study, may promote certain criticism, as there is an uncertain correlation between the PET/CT observations and the real tumor extension, which may only be precisely assessed on the surgical specimen. In the current study, only one case exhibited a cytological correlation with lymph nodes; PET/CT overestimated the lymph node tumor extension. An additional case exhibited a cytological correlation with the primary lesion, however, it was difficult to identify tumor necrosis. FDG uptake was identified in the area of the apparent tumor necrosis, which three of the four observers interpreted as being indicative of tumor. However, the fourth observer interpreted the borderline FDG uptake in the area as not indicating tumor. This difference in interpretation of FDG uptake led to a large degree of interobserver variability in the PET/CT-GTV. The correlation between tumor delineation on PET compared with pathology was investigated by Faria *et al* (29), who compared FDG-PET/CT image fusion with the histopathology of 32 patients. The CT-determined stage was altered by the pathological examination in 22/32 patients (69%), while the PET-determined stage was altered in 16/32 patients (50%). The N-stage was associated with the most significant alterations. The TNM stage was altered by PET in 15/32 patients (44%) compared with CT alone, however, only seven of these alterations were confirmed by the pathological observations.

The consistency of target delineation on PET images is an issue that remains unclear. The literature has shown GTV contouring with FDG-PET to be varied, but these data have typically been based on the SUV. In the present study, an average SUV of ≥2.5 was adopted, consistent with the SUV proposed for lung cancer by another study (30).

The present study shows that FDG-PET/CT images for primary NSCLC exhibit a potential impact on disease staging and treatment planning. A clinical stage variation was observed in 35% of cases (8/23). Based on the results from the present study and from previous literature, the future scenario of the imaging for RT of NSCLC may include the use of functional imaging, such as FDG-PET/CT, with the aim of characterizing the biological features of the tumor and optimizing the use of highly conformal and biologically effective RT.

## Figures and Tables

**Table I:** Characteristics of study population.

Characteristics	Value
Patients, n	23
Age, years	
Median	63
Range	43–76
Gender, n	
Male	19
Female	4
Histology, n	
Squamous cell	16
Adenocarcinoma	7
UICC stage, n	
Ia	1
Ib	1
IIa	1
IIb	3
IIIa	8
IIIb	9

UICC, Union for International Cancer Control.

**Table II:** Change of clinical stage associated with PET/CT in 8/23 patients (35%).

Case, n	CT stage	PET/CT stage
1	T1 N0 M0	T2 N1 M0
2	T4 N2 M0	T3 N3 M0
3	T1 N0 M0	T1 N1 M0
4	T2 N0 M0	T2 N1 M0
5	T2 N0 M0	T2 N1 M0
6	T4N2 M0	T3 N3 M1
7	T4 N2 M0	T2 N2 M1
8	T3 N1 M0	T3 N2 M1

CT, computed tomography; PET/CT, positron emission tomography/CT.

**Table III:** GTV identified by CT and PET/CT in each case.

Patients	GTV-CT, cm^3^	GTV-PET/CT, cm^3^	Ratio
1	19.9	24.3	1.22
2	142.2	158.4	1.11
3	305.2	240.3	1.27
4	30.5	39.7	1.30
5	1.5	1.5	1.00
6	16.1	15.1	1.07
7	40.0	48.3	1.21
8	11.4	12.3	1.08
9	78.0	76.3	1.02
10	31.8	33.4	1.05
11	36.3	59.9	1.65
12	143.7	55.3	2.60
13	155.0	183.9	1.19
14	54.5	62.1	1.14
15	60.1	60.9	1.01
16	79.5	78.7	1.01
17	145.9	167.0	1.14
18	94.8	94.2	1.01
19	122.1	52.6	2.32
20	185.5	62.9	2.95

GTV, gross tumor volume-computed tomography; CT, computed tomography; PET/CT, positron emission tomography/CT.

**Table IV:** Comparison between CT-GTV and PET/CT-GTV for each patient and observer.

	CT-GTV, cm^3^					PET/CT-GTV, cm^3^				
		
Patient	Obs. 1	Obs. 2	Obs. 3	Obs. 4	Ratio	Obs. 1	Obs. 2	Obs. 3	Obs. 4	Ratio
1	27.3	20.3	18.4	12.3	2.22	25.9	20.3	17.4	21.7	1.49
2	152.6	156.4	184.5	137.8	1.34	142.2	158.4	134.5	118.1	1.34
3	349.3	302.2	257.3	58.6	5.96	239.0	242.7	226.4	255.2	1.13
4	44.7	34.1	30.4	36.9	1.47	45.1	34.1	30.4	36.9	1.48
5	1.7	1.4	1.5	1.0	1.70	1.7	1.3	1.5	1.0	1.70
6	16.2	18.1	16.3	35.5	2.19	16.1	21.1	16.3	15.5	1.36
7	59.0	47.3	43.3	39.4	1.50	57.0	48.3	43.3	39.4	1.45
8	11.3	11.3	11.4	11.4	1.01	11.4	11.3	11.5	11.5	1.02
9	78.6	27.3	76.8	50.4	2.88	68.0	98.3	76.8	50.4	1.95
10	34.8	37.4	43.3	32.2	1.34	31.8	33.4	43.3	32.2	1.36
11	75.5	32.5	35.4	44.6	2.32	64.0	57.5	61.5	28.2	2.27
12	224.7	102.3	153.2	65.3	3.44	87.5	107.7	56.1	63.3	1.92
13	145.8	173.9	189.5	143.5	1.32	155.0	183.9	189.5	143.5	1.32
14	67.5	29.2	54.6	52.3	2.31	34.5	56.6	58.6	61.1	1.77
15	80.1	58.9	39.9	56.7	2.01	60.1	60.9	64.9	59.5	1.09
16	232.6	147.0	88.7	65.3	3.56	93.5	78.0	82.4	76.6	1.22
17	120.9	157.0	160.8	123.4	1.33	140.9	157.0	160.8	123.4	1.30
18	71.0	94.2	98.7	86.7	1.39	94.8	73.6	98.7	86.7	1.34
19	180.5	55.2	122.7	43.4	4.16	59.1	69.4	66.7	48.9	1.42
20	186.1	85.9	124.7	68.2	2.73	75.1	60.6	69.4	70.4	1.24

CT-GTV, computed tomography-gross tumor volume; PET/CT-GTV, positron emission tomography/CT-GTV; Obs, observer.
